# Which computer-use behaviours are most indicative of cognitive decline? Insights from an expert reference group

**DOI:** 10.1177/1460458217739342

**Published:** 2017-11-10

**Authors:** Samuel Couth, Gemma Stringer, Iracema Leroi, Alistair G Sutcliffe, Ann Gledson, Davide Bruno, Kathryn R McDonald, Daniela Montaldi, Ellen Poliakoff, Jonathan Rust, Jennifer C Thompson, Laura JE Brown

**Affiliations:** The University of Manchester, UK; Liverpool John Moores University, UK; The University of Manchester, UK; Lancashire Care NHS Foundation Trust, UK; The University of Manchester, UK

**Keywords:** cognitive decline, computer-use activities, dementia, expert opinion

## Abstract

Computer use is becoming ubiquitous among older adults. As computer use depends on complex cognitive functions, measuring individuals’ computer-use behaviours over time may provide a way to detect changes in their cognitive functioning. However, it is uncertain which computer-use behaviour changes are most likely to be associated with declines of particular cognitive functions. To address this, we convened six experts from clinical and cognitive neurosciences to take part in two workshops and a follow-up survey to gain consensus on which computer-use behaviours would likely be the strongest indicators of cognitive decline. This resulted in a list of 21 computer-use behaviours that the majority of experts agreed would offer a ‘strong indication’ of decline in a specific cognitive function, across Memory, Executive function, Language and Perception and Action domains. This list enables a hypothesis-driven approach to analysing computer-use behaviours predicted to be markers of cognitive decline.

## Introduction

The 2016 World Alzheimer’s Report estimated that there were 47 million people worldwide living with dementia, and these numbers are set to increase to 131 million by 2050.^1^ Recent figures suggest that only 40%–50% of people with dementia in high-income countries ever receive a diagnosis and often only then in the moderate or advanced stages of disease progression.^1^ This is important as early diagnosis facilitates interventions that can significantly improve long-term outcomes and patient well-being.^2^

Detecting early cognitive change is challenging for a number of reasons. First, cognitive changes may be subtle, progressive, and/or inconsistent,^3–5^ making them difficult to distinguish from normal variation or age-related changes. This can be further complicated by impairments in memory and self-awareness that often occur in the early stages of dementia.^6–8^ Other factors may also influence people’s willingness to engage with the healthcare system, such as withdrawal, resignation and/or low expectations of care quality.^9^ Furthermore, current methods for detecting dementia are costly due to the specialist time and equipment involved^10^ and only provide a one-off measure of cognitive functioning. Accordingly, there is a real need for low-cost, reliable and unobtrusive methods of continuously gathering and communicating information about individuals’ cognitive functioning so that meaningful decline can be detected and also to reassure cognitively healthy/stable individuals who have concerns about their memory and thinking.

One promising method for detecting subtle cognitive changes over time is by passively measuring everyday computer-use behaviours. That is, continuously collecting data pertaining to an individual’s computer use via recording software, embedded within the individual’s desktop or laptop computer, which does not interfere with normal computer-use operations. Computer use involves many complex activities that depend on a range of cognitive functions (e.g. memory, attention and language). For instance, an everyday task such as finding a saved document requires an individual to remember the name and location of that document and to navigate correctly to it, while also maintaining their attentional focus.^11^ Indeed, recent studies have demonstrated that older adults with mild cognitive impairment (MCI) have reduced and more erratic patterns of computer use,^12,13^ are slower and require more assistance when completing online questionnaires^14^ and are less efficient with their mouse movements^15^ than their cognitively healthy counterparts, suggesting that changes in computer-use behaviours can be indicators of cognitive impairment. Given that levels of computer use in older adults are increasing rapidly,^16,17^ this presents an opportunity to measure computer use to detect subtle cognitive changes in this age group, which may be indicative of a neurodegenerative disorder, such as Alzheimer’s-type dementia.

While continuous tracking of multiple computer-use behaviours has the potential to gather informative data, it also poses a number of challenges. First, little is known about the specific cognitive functions that underlie changes in particular computer-use behaviours, making it difficult to interpret what any changes mean. In addition, the number of computer-use behaviours that could be analysed is potentially vast. Thus, collecting, storing, processing and analysing so much data would require powerful computers with high-end software and hardware; involves producing complex algorithms; is time consuming; and is potentially costly. Moreover, analysing so many variables would greatly increase the odds of falsely detecting a change in computer-use behaviour (type I error).

The ‘Software Architecture for Mental Health Self-Management’ (SAMS) project is a multi-stage research programme investigating whether measuring everyday computer-use behaviours over time may be a pragmatic and sensitive method for detecting early cognitive and functional decline.^11^ As a first step in this project, we aimed to address some of the challenges of understanding and analysing computer-use data by convening a group of experts from clinical and cognitive neurosciences to aid in the selection and interpretation of specific computer-use variables. Using two workshops and a follow-up survey, we aimed to achieve consensus on the following two questions: (1) which patterns of computer-use behaviour are likely to be the most sensitive and specific to detecting early-stage Alzheimer’s-type dementia and (2) which domains of cognitive function are these computer-use behaviours most likely to depend on. The outcomes of this study will help to identify candidate computer-use measures that are likely to be indicators of clinically meaningful cognitive decline.

## Method

Two structured workshops and a follow-up survey were used to gain expert consensus on the identification and interpretation of candidate computer-use behaviours for detecting early-stage Alzheimer’s-type dementia.

### Workshop 1: generating a glossary of relevant cognitive terms

Academic and clinical staff known by the SAMS team to have expertise in the field of cognitive neuroscience or clinical neuropsychology of ageing were invited to attend an initial half-day workshop. The focus was on gaining consensus on the relevance of computer-use data for detecting clinically significant changes in cognition and function. Details of the six invited experts who attended the workshop (authors D.B., K.R.M., D.M., E.P., J.C.T. and J.R.) are detailed in Table 1.

**Table 1.** Expertise and experience of invited attendees of workshops 1 and 2.

Expert	Job title	Years of experience	Qualifications	Sub-speciality
D.B.	Lecturer in Psychology	10	PhD, BSc	Cognitive psychology, cognitive neuroscience, memory, aging and dementia
K.R.M.	Research Associate	8	PhD, MSc, BSc	Cognitive neuroscience of movement disorders and neurodegeneration conditions
D.M.	Professor of Memory Neuroscience	30	PhD, MSc, BA	Cognitive neuroscience, memory function, neuropsychological assessment, dementia
E.P.	Senior Lecturer in Psychology	13	PhD, BSc	Parkinson’s disease, attention, ageing
J.C.T.	Neuropsychologist and Honorary Research Fellow	12	PhD, BSc	Neuropsychology of neurodegenerative disorders
J.R.^a^	Principal Clinical Psychologist	10	PhD, PGDip	Neuropsychology, memory assessment

a Attended workshop 1 only.

Prior to the first workshop, the six experts were provided with a magazine article outlining the broader SAMS project and its objectives.^18^ These aims were also summarised verbally at the start of the workshop, along with the specific objectives of this consensus-gathering study. An exercise was then undertaken to establish an agreed technical vocabulary that could be used to describe cognitive functions in a consistent way throughout the rest of the study. For this, each expert attendee was given a preliminary glossary of 46 specific cognitive terms (e.g. declarative memory, episodic memory, procedural memory) split across six broad domains of cognitive function (e.g. attention, memory, executive function) that had been prepared by the workshop facilitators (authors G.S., I.L. and L.J.E.B) in advance of the workshop using definitions taken from relevant textbooks, handbooks and journal articles. The experts were asked to read through the list and identify any important omissions or points of disagreement with the list of terms or definitions provided. These points were then discussed as a whole group, and the definitions refined to reflect the discussions.

The experts were then asked to consider some common computer-use activities (e.g. logging on, opening a Word document, deleting a folder) and to list the errors or behavioural patterns that they might expect someone with MCI or mild dementia to have with each. They were also asked to consider (from their professional knowledge and clinical experience and using the previously agreed list of cognitive terms), which domains of cognitive function were most likely to be associated with these errors or behavioural patterns. Additional cognitive terms used by attendees during these discussions (e.g. ‘orientation’ and ‘motor control’) were recorded by the workshop facilitators and later added to the glossary of relevant cognitive domains. Further revisions were then made to the glossary after the workshop based on the way that terms had been used by the experts. In the same way, some items (e.g. ‘drawing’ and ‘phonology’) were removed from the glossary when it became clear by their lack of use by the experts that they were not perceived to be relevant to the computer-use tasks. The final glossary contained 31 specific cognitive terms covering six broad domains of cognitive function that were deemed to have some relevance to the changes in computer-use behaviours associated with dementia (available upon request from the corresponding author (I.L.)).

### Workshop 2: consensus agreement on candidate computer-use behaviours for detecting early dementia

Five of the same six experts (plus author (I.L.): a Clinical Senior Lecturer and Consultant in Psychiatry specialising in dementia and member of the SAMS project team) took part in a second workshop approximately 2 months after workshop 1. These six experts were randomly divided into two groups of three, and each group given an identical set of 37 cards that each described a particular change in computer-use behaviour (e.g. errors or slowing of behaviour) associated with common computer-use activities (see, for example, Table 2). The list of computer-use behavioural changes included on the cards had been prepared in advance by the workshop facilitators and was designed to reflect the types of computer-use data that could be collected as part of the broader SAMS project.^11^ The two groups of experts were asked to arrange the cards onto a large sheet of paper labelled with increasing stages of Alzheimer’s-type dementia progression (MCI, Mild, Moderate and Severe) according to where on the disease progression timeline they felt that these behavioural changes would be most likely to first occur. More specifically, ‘MCI’ was the earliest stage of disease progression which was less functionally impactful on computer-use performance than ‘mild AD’, which was less than ‘moderate AD’ and so on, and therefore, certain computer-use operations might only be affected at certain stages of the disease progression. Each group was also given a set of blank cards that they could use to add additional computer-use behaviour changes that they felt were relevant.

**Table 2.** Candidate list of computer-use behaviour changes which were considered most indicative of decline in specific cognitive functions.

Computer-use behaviour change	Cognitive domain	Cognitive term	Level of consensus	Strong indication	Some indication
Sentences are less dense than usual (i.e. uses less verbs, adjectives, adverbs)	Language	Production	Full	100	0
Opens a series of different incorrect folders before opening the correct document in the correct folder	Memory	Recall	Full	100	0
Opens and closes the same wrong Word Document numerous times	Executive function	Inhibition	Full	80	20
Uses a reduced set of vocabulary in emails	Language	Production	Full	80	20
Repeatedly types a series of different incorrect passwords (e.g. Dog1, Cat1, Dog2) after receiving ‘incorrect username/password’ messages	Memory	Declarative	Full	80	20
Opens a series of incorrect Word documents before opening the correct document	Memory	Recall	Full	80	20
Repeatedly types a series of different incorrect passwords (e.g. Dog1, Cat1, Dog2) after receiving ‘incorrect username/password’ messages	Memory	Recall	Full	80	20
Clicks the mouse more than five times in rapid succession on the programme icon	Perception and action	Motor control	Full	80	20
Repeatedly double clicks in areas of the screen that are close to (but not on) the programme icon	Perception and action	Spatial perception	Full	80	20
Repeatedly types the same incorrect password (e.g. Dog1, Dog1) despite receiving ‘incorrect username/password’ messages	Executive function	Self-error monitoring	Full	60	40
Sentences are shorter than usual	Language	Production	Full	60	40
Computer-use behaviour	Cognitive domain	Cognitive term	Consensus	Strong indication	Some indication
Repeatedly types the same incorrect password (e.g. Dog1, Dog1) despite receiving ‘incorrect username/password’ messages	Memory	Declarative	Full	60	40
Opens a series of different incorrect folders before opening the correct document in the correct folder	Memory	Declarative	Moderate	80	0
Clicks the mouse more than five times in rapid succession on the programme icon	Executive function	Inhibition	Moderate	60	20
Repeatedly makes single clicks on the programme icon despite the programme not opening	Executive function	Inhibition	Moderate	60	20
Opens and closes the same wrong Word document numerous times	Executive function	Self-error monitoring	Moderate	60	20
Opens a series of incorrect Word documents before opening the correct document	Memory	Declarative	Moderate	60	20
Repeatedly types the same incorrect password (e.g. Dog1, Dog1) despite receiving ‘incorrect username/password’ messages	Memory	Recall	Moderate	60	20
Completes the email but does not send, that is, left as a draft	Memory	Short term/working memory	Moderate	60	20
Opens the same incorrect folder numerous times without opening a Word document	Memory	Short term/working memory	Moderate	60	20
Repeatedly types the same incorrect password (e.g. Dog1, Dog1) despite receiving ‘incorrect username/password’ messages	Memory	Short term/working memory	Moderate	60	20

The two groups then came together to discuss the decisions that they had made and the reasons behind them. Each group was also asked to select between 5 and 10 cards that they felt would best meet the criteria of being: (1) early predictors of, (2) most sensitive to and (3) most specific to Alzheimer’s-type dementia. The two groups then compared the sets of cards that they had each chosen and, through discussion, agreed on 10 cards that they felt best met the criteria, as well as being most pragmatic and relevant to the majority of computer users.

The experts were then randomly split into two different groups of three, given a second set of 31 cards that described a different set of changes in computer-use behaviour. They were asked to organise the cards horizontally onto the disease timeline according to when the change was most likely to first occur and vertically according to the sensitivity and specificity of the change as an indicator of Alzheimer’s-type dementia. Each group was then asked to select 5–10 cards that best met the same three criteria as before and then came together to discuss their respective choices. On the basis of this discussion, the experts agreed on seven cards from the original pack, plus an additional five cards describing activities that they had generated themselves. A total of 22 computer-use behaviours were therefore selected from this workshop as being candidates for detecting early Alzheimer’s-type dementia. This list is available upon request from the corresponding author (I.L.).

### Survey: linking relevant computer-use behaviours to underlying cognitive domains

Following workshop 2, a copy of the glossary of the 31 cognitive terms from workshop 1, and a survey linking the cognitive terms to the computer-use behaviours selected in workshop 2, was sent by mail to each of the six experts from workshop 1. The survey listed each of the 22 computer-use behaviours against columns containing the 31 cognitive terms (across six broad cognitive domains), resulting in a total of 682 cognition-behaviour combinations. For each combination, the experts were instructed to indicate the extent to which the specified behaviour could be an indicator of impairment in a particular cognitive function by giving one tick if they thought it would provide ‘Some indication’, two ticks for a ‘Strong indication’ and leaving it blank if they thought it would provide ‘No indication’.

## Results

Five out of the six surveys were completed and returned. In order to determine which computer-use behaviours were reliably considered to be likely indicators of cognitive change, levels of consensus between the experts (i.e. amount of agreement regarding whether each behaviour did or did not indicate impairment in a specific cognitive function) and the strength of the indication (none, some or strong) were calculated. When considering consensus, responses were dichotomised into reflecting any indication (i.e. ‘Strong’ or ‘Some’ indication responses) versus ‘No indication’. Full consensus was thus defined as either all five experts responding that a behaviour gave ‘Some’ or ‘Strong’ indication of impairment in that cognitive function, or all five responding that it gave ‘No indication’. Moderate consensus was defined as four of five experts responding in one of these ways. Occasions where there was a conflict in agreement, that is, three of five responding with ‘Some’ or ‘Strong’ indication, or three of five responding with ‘No indication’, were deemed as no consensus (for review on consensus measurement in Delphi-style designs, see the work by von der Gracht^19^). To determine only the most relevant behavioural indicators of cognitive change, we focused on items where at least moderate consensus was reached and where at least three experts had indicated ‘Strong indication’.

### Consensus

**Figure 1.**
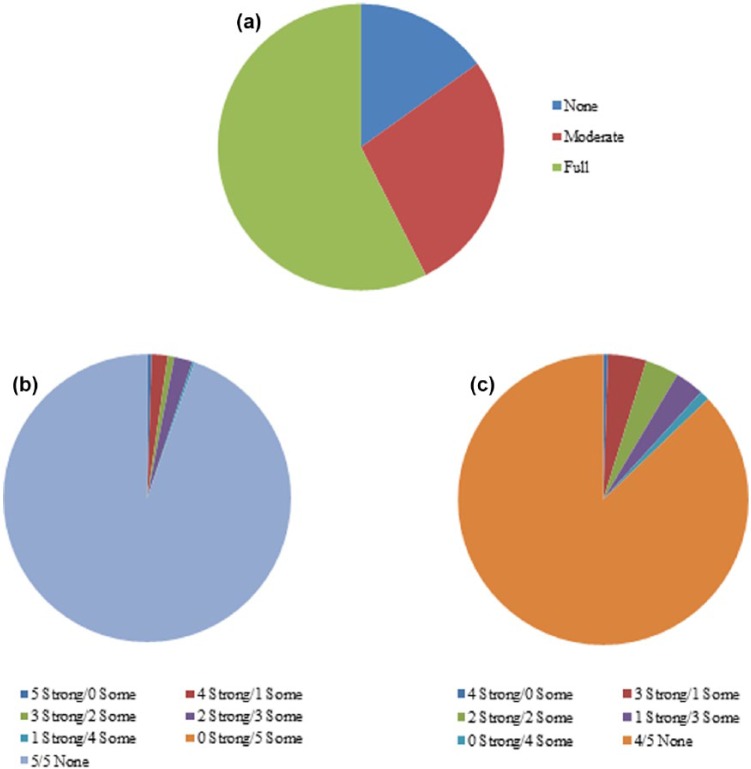
(a) Percentage full, moderate and no consensus overall. Level of indication (i.e. Strong/Some/None) for (b) full consensus and (c) moderate consensus.

Full consensus was achieved for 392/682 (57.5%) of the cognition–behaviour combinations (Figure 1(a)). Of this, experts responded with ‘Some’ or ‘Strong’ indication for 21 (3.08% overall/5.36% full) combinations and ‘No indication’ for 371 (54.4% overall/94.6% full) combinations (Figure 1(b)). Moderate consensus was achieved for 187/682 (27.4%). Of this, experts responded with ‘Some’ or ‘Strong’ indication for 24 (3.52% overall/12.8% moderate) combinations and ‘No indication’ for 163 (23.9% overall/87.2% moderate) combinations (Figure 1(c)). Overall, this indicated that the experts agreed on the involvement (or non-involvement) of specific cognitive functions for specific computer-use behaviours in the majority of cases, that is, moderate consensus or higher for 579/682 (84.9%) of the combinations.

### Strongest indicators

There were 21 (3.08% overall) cognition–behaviour combinations for which there was full or moderate consensus and with at least three of these experts indicating ‘Strong indication’. Collectively, this formed the list of computer-use behaviour changes which were likely to be indicative of decline in specific cognitive functions (Table 2). These 21 items covered Memory, Executive function, Language and Perception and Action cognitive domains.

### Computer-use behaviours

Of the 22 computer-use behaviours produced in workshop 2, full consensus on underlying cognitive functions was reached for 15, moderate consensus for 4 and no consensus for 3. The strongest cognition–behaviour combination(s) (i.e. highest consensus and strongest indication) for each of the 22 computer-use behaviours is available upon request. This includes impairments from five of six broad cognitive domains, with the exception of processing speed.

## Discussion

The aim of this study was to produce a list of candidate computer-use behaviour changes that are likely to be the most sensitive and specific to detecting the cognitive changes associated with early-stage Alzheimer’s-type dementia. Focusing on these behaviours when measuring older adults’ computer use would reduce the time and costs associated with collecting, storing and analysing a vast data set of computer behaviours, as well as the chances of finding false positives. Experts from clinical and cognitive neurosciences were convened to take part in two workshops and a survey, which resulted in the identification of 21 computer-use behaviours that could be further tested for sensitivity and specificity for early Alzheimer’s-type dementia, as well as specific computer-use behaviours that might be indicative of decline to Memory, Executive function, Language and Perception and Action.

Previous studies investigating computer-use behaviours have focused on behaviours that could be considered to be simpler, more routine behaviours, such as mouse movement coordination.^15^ In contrast, the candidate list of behaviours reported here are more operationally complex and cover a broader range of computer-use behaviours.^11^ For example, the experts identified errors in computer-use operations, such as opening incorrect folders and typing incorrect passwords, as well as decline in functional activities conducted via the computer, such as typing shorter and less dense sentences (see Table 2). An advantage of these latter type of behaviours is that they are not necessarily restricted to desktop or laptop computers or to one type of computer function (i.e. mouse movements) and could be applied to new and evolving technologies (e.g. tablets and smartphones).

While these complex behaviours may be more difficult to extract and interpret objectively through passive monitoring, they arguably offer greater insight into impairment in specific cognitive domains. For instance, Seelye et al.^15^ showed correlations between mouse movement coordination and numerous broad cognitive domains including executive functioning, attention, visual–spatial and ‘global cognition’. In contrast, our candidate list comprises 13 separate computer-use behaviours that are each considered to be particularly indicative of one (or more, see below) of eight specific cognitive functions. For example, the experts identified ‘Repeatedly makes single clicks on the programme icon despite the programme not opening’ as being specifically related to inhibition (Executive function); thus, this behaviour might be useful for detecting impairment that is exclusive to this cognitive function. When considered collectively, an individual’s pattern of behavioural changes could therefore prove useful for characterising their specific disease profile. For instance, although this candidate list was produced with Alzheimer’s-type dementia in mind, some of the candidate behaviours were related to cognitive functions associated with other types of dementia and so could aid with differential diagnosis. For example, frontotemporal dementia is characterised by reduced inhibition,^20^ which is a cognitive domain that the experts associated with three of the computer-use behaviours.

Some candidate behaviours were related to impairments in multiple cognitive domains. For example, ‘Repeatedly types the same incorrect password (e.g. Dog1; Dog1…) despite receiving “incorrect username/password” messages’ was related to Short-term/Working memory, Memory recall, Declarative memory and Self-error monitoring. Although these behaviours might be less useful for identifying specific patterns of change, these ‘broad brush’ behaviours might be more sensitive to detecting cognitive decline more generally (as per the work by Seelye et al.^15^). Therefore, it might be beneficial to examine a number of computer-use behaviours that can detect cognitive decline more generally in the first instance and then to focus on some of the more specific behaviour–cognition pairings to more precisely determine which cognitive domains are affected.

Note that there may be a number of reasons why computer-use behaviours might be affected other than cognitive decline, such as fatigue, mood, task complexity/novelty and environmental distractors. Accordingly, without knowing all of these precise contextual features, it is not possible to definitively identify or diagnose cognitive decline from passive monitoring alone. Nevertheless, if there was a steady change in computer-use performance from an individual’s initial ‘baseline’ ability, it may be possible to identify some degree of cognitive decline as opposed to fluctuations in functioning due to uncontrollable factors and could therefore be used to refer the individual to their clinician for further assessment. A more in-depth analysis of specific computer-use behaviours could then be used to supplement, rather than replace, existing clinical measures for diagnosing the exact type of dementia.

There are some limitations to this study. First, our list of computer-use behaviours is not definitive and there are many other computer-use behaviours that could have been included. However, as part of the study, the experts could, and did, add some of their own suggestions to this list, which reduced the chance of clinically important computer-use behaviours being omitted. A second issue relates to the fact that, as computer-use habits will differ between individuals, not all of the candidate behaviours listed will be relevant to every user. For instance, some people may use a computer to write emails but never to save or retrieve files. This underlies the importance of considering a variety of behaviours that relate to several different computer operations in order to maximise the chances that at least some of the behaviours will be relevant to an individual.

Another limitation is that the technical terms, computer-use behaviours and the candidate list are based on the opinions of a small number of experts who completed the workshops and the survey. While the inclusion of more experts could have revealed more diverse and valid insights, having a small group ensured that the focus remained on the topic and the task at hand, thus enabling more meaningful discussions. Additionally, all of the experts who completed the survey had attended at least one of the study workshops and had spent time discussing and thinking about the issues, which means that they were very well informed about the aims of the project for completing the survey. Indeed, this would explain the reliability in the responses; consensus was reached for a large number of the computer-use behaviour–cognitive domain pairings (84.9% of moderate consensus or higher), and thus, we were able to produce a select list of the most specific computer-use behaviours.

One important consideration which was not included in this study is the involvement of patients (i.e. the end-user), informants and the public in the development of health monitoring software.^21–23^ One of the preliminary aims of the SAMS project was to determine the acceptability of passive recording measures for monitoring mental health among these individuals,^24^ and further input will be required on the design and usability of the recording software interface. However, it may not have been appropriate to include these individuals at the stage of the project presented here, whereby it was essential to gather opinions from experts in clinical and cognitive neuroscience who have a detailed knowledge and understanding of specific cognitive terms (e.g. executive functioning, declarative memory, processing speed) and how these might be related to different behaviours.

In conclusion, our group-based approach using experts (similar to Delphi-type methods, e.g.)^25^ across clinical and cognitive neuroscience was useful for determining which computer-use behaviour changes may be ideal candidates for detecting cognitive decline. Being able to interpret daily computer-use behaviours may provide an ecologically valid assessment based on an increasingly common daily task that depends on a complex interaction of higher cognitive functions such as executive function. This may help to define cognitive-related functional decline which will facilitate earlier and more accurate diagnosis, thereby addressing the clinical needs of people with cognitive impairment. This approach also highlighted other areas of complexity and considerations when monitoring computer-use behaviours as a proxy measure of dementia, as well as some valuable insights to workshop and survey-based methods. The next phase of the SAMS study aims to validate these candidate computer-use behaviours empirically and to determine their sensitivity and selectivity for early dementia.
